# A Ferrocene-Quinoxaline Derivative as a Highly Selective Probe for Colorimetric and Redox Sensing of Toxic Mercury(II) Cations

**DOI:** 10.3390/s101211311

**Published:** 2010-12-10

**Authors:** Fabiola Zapata, Antonio Caballero, Pedro Molina, Alberto Tarraga

**Affiliations:** Departamento de Quimica Organica, Facultad de Quimica, Universidad de Murcia, Campus de Espinardo, E-30100 Murcia, Spain; E-Mails: fazafer@um.es (F.Z.); antocaba@um.es (A.C.)

**Keywords:** ferrocene, quinoxaline, mercury, electrochemistry, UV-vis spectroscopy

## Abstract

A new chemosensor molecule **3** based on a ferrocene-quinoxaline dyad recognizes mercury (II) cations in acetonitrile solution. Upon recognition, an anodic shift of the ferrocene/ferrocenium oxidation peaks and a progressive red-shift (Δλ = 140 nm) of the low-energy band, are observed in its absorption spectrum. This change in the absorption spectrum is accompanied by a colour change from orange to deep green, which can be used for a “naked-eye” detection of this metal cation.

## 1. Introduction

The design and synthesis of chemosensors for environmentally and biologically relevant species have been actively investigated in recent years [1–3]. In this regard, chemosensors that can highly sensitively and selectively monitor heavy metal ions are especially important. Among heavy and transition metals, mercury, widely distributed in air, water and soil, is considered to be one of the highly toxic because both elemental and ionic mercury can be converted by bacteria in the environment to methyl mercury, which subsequently bioaccumulates through the food chain [4–11]. Mercury-induced toxicity can cause a number of severe health problems because it can damage the digestive organs, kidneys, central nervous system and endocrine system [12–17]. Given its high toxicity and the increasing threat of global mercury release into the environment, considerable efforts are continuously made to develop highly selective and sensitive chemosensors for Hg(II). In this context, development of new and practical chemosensors which offer a promising approach for mercury ion detection is still a great challenge for the scientific community [18–23], triggering a large number of related investigations that have been recently reviewed [24–26].

Ferrocene is one of the favourite “building blocks” in the construction of sensing platforms based on redox-active units due to the availability, stability and tailorability of most of its derivatives. The sensing behaviour of these systems is mainly based on the potential shift shown upon their interaction with a variety of guest species. However, binding can also affect the UV-vis properties of the ferrocene unit when it is placed near the binding site. In general, metal complexation induces bathochromic shifts in the lower-energy, spin-allowed ferrocene absorption band, which is between 400 and 500 nm [27–30]

On the other hand, quinoxaline derivatives are the subject of considerable interest from both academic and industrial perspectives because they are significant intermediates for the manufacture of pharmaceuticals and advanced materials [31–34] Moreover, the quinoxaline ring appropriately subtitued or fused to some other azaheterocyclic systems has also been studied as a putative binding subunit for the recognition and sensing of both anionic and cationic especies [35–37]

The work presented here, forms part of our interest in designing chemosensors that are capable of reporting on the recognition of metal cations through a variety of physical responses, by combining various signalling units into an individual molecule. Toward this end, we report here a straightforward synthesis of the new 2,3-diferrocenylquinoxaline ligand which shows a selective, sensitive and reversible response to the Hg(II) ion through two different channels: redox and chromogenic

## 2. Experimental Section

All reactions were carried out using solvents which were dried by routine procedures. The melting point was determined on a hot-plate melting point apparatus and is uncorrected. ^1^H- and ^13^C-NMR spectra were recorded at 400 and 100 MHz, respectively on a Brucker AC 400. The following abbreviations for stating the multiplicity of the signals have been used: s (singlet), bs, d (doublet), t (triplet), st (pseudotriplet), and q (quaternary carbon atom). Chemical shifts refer to signals of tetramethylsilane in the case of ^1^H- and ^13^C-NMR spectra. The cyclic electrochemistry measurements were performed on a Bioanalytical Systems CV-50 W Voltammetric Analyzer potentiostat/galvanostat controlled by a personal computer and driven by dedicated software with a conventional three-electrode configuration consisting of platinum working and auxiliary electrodes and an SCE reference electrode. The experiments were carried out with a 10^−3^ M solution of sample in dry CH_3_CN containing 0.1 M [(*n*-Bu)_4_N]ClO_4_ as supporting electrolyte (***Warning****: Potential formation of highly explosive perchlorate salts of organic derivatives*). Deoxygenation of the solutions was achieved by bubbling nitrogen for at least 10 min, and the working electrode was cleaned after each run. The cyclic voltammograms were recorded with a scan rate between 0.05 and 0.5 V s^−1^. Linear sweep voltammetry (LSV), cyclic voltammetry (CV), and Osteryoung square wave voltammetry (OSWV) were recorded before and after the addition of aliquots of 0.1 equiv of 2.5 × 10^−2^ M solutions of the corresponding cations in H_2_O. The following settings were used: pulse amplitude, 50 mV; pulse width, 50 ms; scan rate, 100 mV/s; sample width, 17 ms; pulse period, 200 ms. Decamethylferrocene (DMFe) (−0.07 V *vs* SCE) was used as an internal reference both for potential calibration and for reversibility criteria. UV-vis absorption spectra were regularly recorded after the addition a small aliquot of the corresponding cation (c = 2.5 × 10^−3^ M) to a solution of the receptor (c = 1 × 10^−4^ M) using a UV quartz cell.

### 2.1. Preparation of 2,3-diferrocenylquinoxaline (**3**)

2,3-Diaminobenzene (**1**, 77 mg, 0.7 mmol) was added to a solution of diferrocenylethane-1,2-dione (**2**, 0.3 g, 0.7 mmol) in ethanol (50 mL). The mixture was stirred under reflux overnight during which time an orange solid precipitated, which was isolated by filtration, washed with cold diethyl ether (3 × 10 mL) and finally crystallized in ethanol. Yield 98%. M.p > 300 °C. ^1^H-NMR (CD_3_CN): δ 4.09 (s, 10H), 4.32 (st, 4H), 4.64 (st, 4H), 7.67 (dd, 2H, *J* = 3.4 Hz, *J* = 6.4 Hz), 8.03 (dd, 2H, *J* = 3.4 Hz, *J* = 6.4 Hz); ^13^C-NMR (CDCl_3_): δ 68.7 (4xCH), 69.7 (10xCH), 71.4 (4xCH), 85.2 (2xq), 128.5 (2xCH), 128.7 (2xCH), 140.4 (2xq), 152.9 (2xq); FAB MS: *m/z* (relative intensity): 498 (M^+^,100); Anal Calc for C_28_H_22_Fe_2_N_2_: C, 67.57; H, 4.45; N, 5.62. Found: C, 67.80; H, 4.82; N, 5.40.

## 3. Results and Discussion

### 3.1. Synthesis

**Scheme 1.**
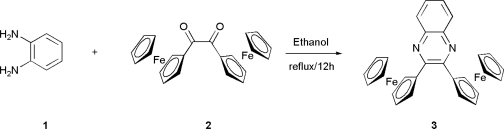
Preparation of receptor **3**.

The quinoxaline-based receptor **3** was prepared following the classical method for synthesizing both quinoxaline itself and its derivatives, which involves the condensation of an aromatic 1,2-diamine with a 1,2-dicarbonyl compound in refluxing ethanol or acetic acid (Scheme 1) [38]. Thus, condensation of the readily available diferrocenylethane-1,2-dione (**2)** [35] with 1,2-diaminobenzene (**1**) gave an excellent yield (98%) of the corresponding 2,3-diferrocenylquinoxaline (**3**) which was fully characterized by using standard techniques: ^1^H-NMR and ^13^C-NMR spectroscopies, FAB mass spectrometry and elemental analysis.

### 3.2. Electrochemical and Optical Properties

**Figure 1.**
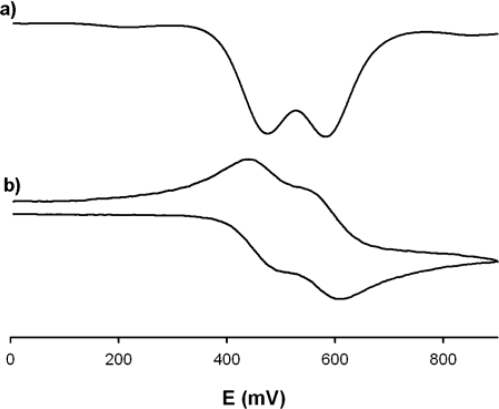
OSWV (**a)** and CV (**b)** of receptor **3** (1mM) in CH_3_CN using [*n*-Bu_4_N]ClO_4_ as supporting electrolyte scanned at 100 mV/s.

The redox properties of receptor **3** was investigated by linear sweep voltammetry (LSV), cyclic voltammetry (CV), and Osteryoung square wave voltammetry (OSWV) in a CH_3_CN solution containing 0.15 M [*n*-Bu_4_N]ClO_4_ (TBAP) as supporting electrolyte. In spite of the symmetry of the receptor **3** it exhibited, in the range 0−0.9 V, two reversible one-electron redox wave at the half-wave potential value of *^1^E_1/2_* = 0.47 V and *^2^E_1/2_* = 0.58 V (Δ*E_1/2_* = 110mV) versus decamethylferrocene (DMFc), demonstrating the existence of a weak interaction between the two iron centres (Figure 1). The criteria applied for reversibility was a separation of ∼60 mV between cathodic and anodic peaks, a ratio of 1.0 ± 0.1 for the intensities of the cathodic and anodic currents Ic/Ia, and no shift of the half-wave potentials with varying scan rates.

The UV−vis spectra for receptor **3** was recorded as 10^−4^ M solution in CH_3_CN and contains three prominent absorption bands with a maximum at 234 nm (ɛ = 26,000 M^−1^ cm^−1^), 277 nm (ɛ = 14750 M^−1^ cm^−1^) and 314 nm (ɛ = 9420 M^−1^ cm^−1^) which can safely be ascribed to a high energy ligand-centered π−π* electronic transition (L−π*) (HE band). In addition to this band, another two weaker absorptions are visible at 409 nm (ɛ = 1,590 M^−1^ cm^−1^) and 490 nm (ɛ = 1,860 M^−1^ cm^−1^) which are assigned to another localized excitations with a lower energy produced either by two nearly degenerate transitions, an Fe(II) d−d transition or by a metal−ligand charge transfer (MLCT) process (d_π_−π*) (LE band) [39] This assignment is in accordance with the latest theoretical treatment (model III) reported by Barlow *et al.* [40]. Such spectral characteristics confer an orange color to this species.

### 3.3. Cation Sensing Properties

One of the most interesting attributes of the new diferrocenylquinoxaline reported here is the presence of metal-ion binding sites on the quinoxaline ring close to a ferrocene redox-active moiety. Due to this structural feature metal recognition properties on the receptor **3** were evaluated by electrochemical, optical and ^1^H-NMR techniques.

**Figure 2.**
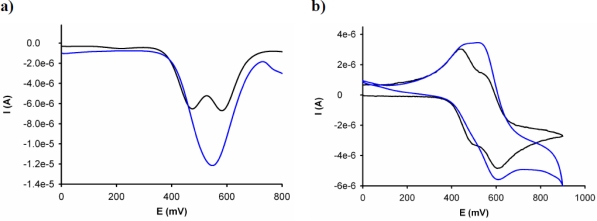
Evolution of the OSWV (**a)** and CV (**b)** of **3** (c = 1 × 10^−3^ M in CH_3_CN) (black line) with the addition of 1 equiv of Hg^2+^ cations in H_2_O (blue line), using TBAP as supporting electrolyte, scanned at 0.1 V/s.

The electrochemical binding interactions of **3** towards cations of biological and environmental relevance, such as Li^+^, Na^+^, K^+^, Ca^2+^, Mg^2+^, Cu^2+^, Zn^2+^, Cd^2+^, Hg^2+^, Ni^2+^, and Pb^2+^, added as their perchlorate salts, were investigated in CH_3_CN (c = 1 × 10^−3^ M). Titration studies with addition of the above-mentioned set of metal cations (2.5 × 10^−2^ M in H_2_O) to an electrochemical solution of receptor **3** containing [*n*-Bu_4_N]ClO_4_ (0.1 M) as supporting electrolyte, demonstrate that while addition of Cu^2+^ and Hg^2+^ ions promotes remarkable responses, addition of Li^+^, Na^+^, K^+^, Ca^2+^, Mg^2+^, Zn^2+^, Cd^2+^, Pb^2+^ and Ni^2+^ metal ions had no effect either on LSV or on the CV or OSWV of this receptor, even when present in a large excess. The results obtained on the stepwise addition of substoichiometric amounts of Hg^2+^ revealed the appearance, in the OSWV, of a new oxidation peak at practically the same potential of the second redox peak in the free receptor (*Ep* = 0.55 V, *ΔEp* = 75 mV).This fact suggests that the complex is disrupted after the first monoelectronic oxidation of the complex **3**^+^·Hg^2+^ and the second oxidation really takes place on the uncomplexed mono-oxidized **3**^+^. The current intensity of this new peak increases until 1 equiv of the Hg^2+^ cation is added [Figure 2(a)]. Moreover, the CV analysis of the complex **3**·Hg^2+^ shows that one reduction process takes place at the same reduction potential showed by the uncomplexed ligand **3**, indicating that the complex starts to be disrupted after its electronic oxidation [Figure 2(b)]. This behaviour means that this receptor is not only able to monitor binding but it is also able to behave as an electrochemically induced switchable chemosensor for Hg^2+^ through the progressive electrochemical release of these metal cations; as a result of a decrease of the corresponding binding constant upon electrochemical oxidation.

**Figure 3.**
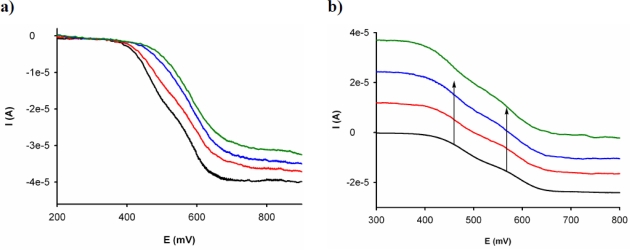
Changes in the LSV of **3** (1 × 10^−3^ M in CH_3_CN) (black line) with the addition increasing amounts until 1 equiv of Hg^2+^ (a) and Cu^2+^ (b) cations in H_2_O (green line) using TBAP as supporting electrolyte and a rotating disk electrode at 100 mV/s and 1,000 rpm.

Remarkably, LSV studies carried out upon addition of Cu^2+^ to the CH_3_CN solution of this receptor showed a significant shift of the sigmoidal voltammetric wave toward cathodic currents, indicating that Cu^2+^ cations promote the oxidation of the free receptor. On the other hand, the same experiments carried out upon addition of Hg^2+^ revealed a shift of the linear sweep voltammogram toward more positive potentials, indicating the complexation process according to the previously observed by OSWV (Figure 3).

Previous studies on ferrocene-based ligands have shown that their characteristic low energy (LE) bands in the absorption spectra are perturbed upon complexation [41–44]. Therefore, the metal recognition properties of the ligand **3** toward metal ions were also evaluated by UV−vis spectroscopy. Titration experiments for CH_3_CN solutions of this ligand (c = 1 × 10^−4^ M), and the corresponding cations were performed and analyzed quantitatively. [45] It is worth mentioning that no changes were observed in the UV−vis spectra upon addition of Li^+^, Na^+^, K^+^, Ca^2+^, Mg^2+^, Zn^2+^, Cd^2+^, and Ni^2+^ and Pb^2+^ metal ions, even in a large excess; however, significant modifications were observed upon addition of Hg^2+^.

**Figure 4.**
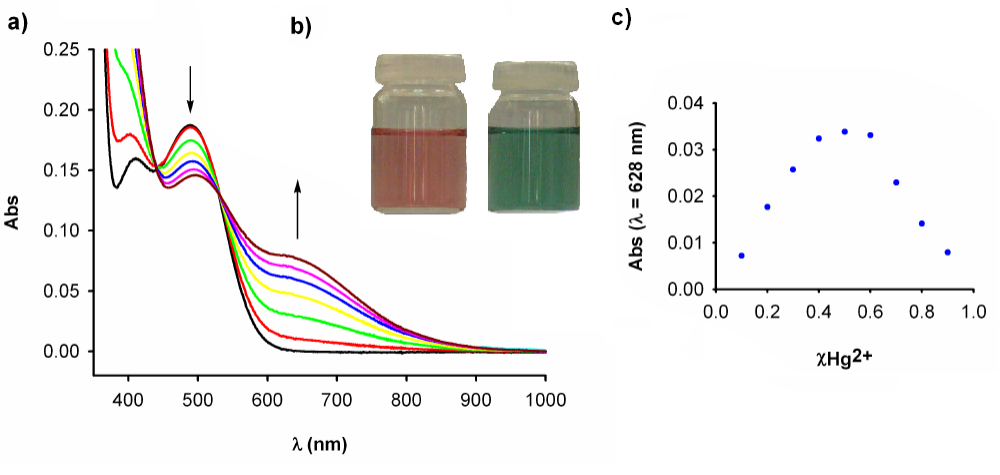
(a) Changes in the absorption spectra of **3** (c = 1 × 10^−4^ M in CH_3_CN) (black line) with the addition increasing amounts of Hg^2+^ in water until 1 equiv; arrows indicate the absorptions that increase or decrease during the experiment. (b) Changes in the color of receptor **3** (left) upon addition of Hg^2+^ cations (right). (c) Job’s plot for **3** and Hg^2+^, indicating the formation of a 1:1 complex; the total [**3**] + [Hg^2+^] = 1 × 10^−4^ M (λ_abs_ = 628 nm)

Thus, the addition of increasing amounts of Hg^2+^ ions in water to a solution of **3** caused a decrease in the intensity of the LE band, at λ = 490 nm, along with the progressive appearance of a new band located at λ = 630 nm (ɛ = 790 M^−1^ cm^−1^) as well as a increase of the initial HE band intensity. Two well-defined isosbestic points at 439 and 531 indicate that a neat interconversion between the uncomplexed and complexed species occurs [Figure 4(a)]. The new LE band is red-shifted by 140 nm and is responsible for the change of colour, from orange to deep green, which can be used for a “naked-eye” detection of this metal ion [Figure 4(b)]. Binding assays using the method of continuous variations (Job’s plot) suggests a 1:1 binding model (metal/ligand) with a log *Ka* = 3.4 ± 0.17 [Figure 4(c)]. Moreover, the calculated detection limit [46] was 1.3 × 10^−5^ M. Additionally the peak corresponding to the complex [**3·**Hg]^2+^ was observed by ES-MS at *m*/*z* 700.02. The relative abundance of the isotopic clusters was in good agreement with the simulated spectrum of the 1:1 complex.

**Figure 5.**
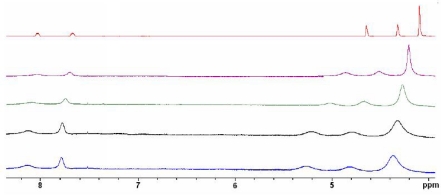
^1^H-NMR spectral changes observed in **3** (red) in CD_3_CN (red line) during the addition of up to 1 equiv of Hg^2+^ in D_2_O (blue line).

In order to get additional information about the coordination between the receptor **3** and Hg^2+^ cations, a ^1^H-NMR titration experiment was performed where aliquots of metal cation in D_2_O were added to a solution of the receptor in CD_3_CN. The free receptor **3** exhibits two sets of signals: one of them corresponding to the ferrocene moiety and another one to the quinoxaline ring. The ferrone moieties show a signal at δ = 4.10 (s), corresponding to the protons present in the unsubstituted ciclopentadienyl (Cp) unit and two psudotriplets at 4.32 and 4.64 ppm assigned to the H_β_, and H_α_ within the monosubustituted Cp ring. On the other hand, the quinoxaline ring displays two double doublets at δ = 7.67 (H-6) and 8.03 (H-5) ppm. An inspection of the ^1^H-NMR titration data showed a strong chemical shift for the signals associated with the ferrocene units due to their proximity to the binding sites. The protons within the unsubstituted Cp were shifted Δδ = +0.26 ppm and the H_α_ and H_β_ protons Δδ = 0.59 and 0.52 ppm respectively. On the other hand a weaker shift (Δδ = 0.1 ppm) in the H-5 and H-6 protons of the quinoxaline ring were also observed (Figure 5).

## 4. Conclusions

We have successfully developed a new and easy-to-make quinoxaline-based molecular sensor **3** which shows selective response to Hg^2+^ ions through a dual channel: Electrochemical and chromogenic. The reported quinoxaline-ferrocene sensor permits not only the naked-eye detection of this metal cation but also to monitor the recognition process through electrochemical measurements. Additionally, this receptor is also able to behave as an electrochemically induced switchable chemosensor for Hg^2+^. A combination of the UV-vis titration data and mass spectrometry has been successfully used to establish the 1:1 stoichiometry of the complex formed.
